# Setting research priorities for management and treatment of hyperhidrosis: the results of the James Lind Alliance Priority Setting Partnership

**DOI:** 10.1111/ced.15122

**Published:** 2022-03-04

**Authors:** Louise Jane Dunford, Kathy Radley, Margaret McPhee, Louise McDonald, Richard John Oliver, Anton Alexandroff, Hana Amber Hussain, Janice Adasa Miller, Maryrose Tarpey, Andrew Victor Clifton

**Affiliations:** ^1^ Institute of Allied Health Sciences De Montfort University Leicester UK; ^2^ Centre for Postgraduate Medicine and Public Health University of Hertfordshire Hatfield Hertfordshire UK; ^3^ UK Dermatology Clinical Trials Network University of Nottingham Nottingham UK; ^4^ Department of Dermatology Ulster Hospital Belfast UK; ^5^ Hyperhidrosis UK Hereford UK; ^6^ James Lind Alliance, The Wessex Institute University of Southampton Southampton Hampshire UK; ^7^ School of Nursing Midwifery De Montfort University Leicester UK

## Abstract

**Background:**

Hyperhidrosis is a common skin condition characterized by excessive sweating, which can negatively impact on quality of life. It is under‐researched compared with other conditions of similar prevalence.

**Aim:**

To generate a Top 10 list of research priorities for the treatment and management of hyperhidrosis, with equal input from people with hyperhidrosis and healthcare professionals (HCPs).

**Methods:**

A priority setting partnership (PSP) was established and processes from the James Lind Alliance Handbook were followed. An online survey asked participants what questions they would like research to answer. These questions were grouped into ‘indicative questions’, which were ranked in a second survey of 45 indicative questions. The top 23 questions were then taken to a final workshop event attended by key stakeholders, and ranked to generate the Top 10 list of research priorities.

**Results:**

There were 592 questions submitted by 268 respondents for the first survey. For the second survey, 286 participants ranked the indicative questions in order of priority. At the final workshop, the Top 10 list was generated. The top three priorities were: (i) Are there any safe and effective permanent solutions for hyperhidrosis? (ii) What is the most effective and safe oral treatment (drugs taken by mouth) for hyperhidrosis? and (iii) What are the most effective and safe ways to reduce sweating in particular areas of the body?

**Conclusions:**

There are many unanswered research questions that both people with hyperhidrosis and HCPs would like to see answered. The results from this PSP will help to ensure future research funding can be directed to these areas of priority.

## Graphical abstract

A priority setting partnership (PSP) between people with hyperhidrosis and healthcare professionals was set up to generate the Top 10 list of research priorities for management and treatment of hyperhidrosis. The PSP followed James Lind Alliance methodology.

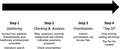

## Introduction

Hyperhidrosis is a common skin condition characterized by abnormal levels of sweating beyond physiological need. Prevalence ranges from 1 to 5% worldwide, and it affects both sexes equally.^1^, ^2^ Hyperhidrosis can be categorized as primary (idiopathic) or secondary to many other conditions.^3^ Primary hyperhidrosis often starts in childhood or at puberty,^2^ and the most commonly affected areas are hands, feet, underarms, face/head or groin.^4^ Hyperhidrosis can have a significant negative impact on quality of life (QoL), causing both physical problems and psychological distress. Many people with hyperhidrosis are embarrassed to seek medical help, and only half ever discuss their condition with a healthcare professional (HCP).^5^ When patients with hyperhidrosis do seek medical help, it can be hampered by poor clinical guidelines and a lack of scientific evidence.^6^, ^7^

A wide range of interventions are available for hyperhidrosis, including topical treatments, injectables, oral anticholinergics and destructive treatments. However, the efficacy of treatments is often limited or they have adverse effects.^8^ Compared with other skin disorders of similar prevalence, hyperhidrosis research is less well funded. Currently there are only 9 clinical trials for hyperhidrosis recruiting worldwide, whereas psoriasis, with a similar prevalence, has 193.^9^

Priority setting partnerships (PSPs) enable patients and HCPs to work together as equal partners to identify questions about treatments that are not currently answered by existing research. This PSP aimed to identify and prioritize the Top 10 most important research uncertainties relating to management and treatment of hyperhidrosis, to ensure that funding is directed to the areas of research that matter most to patients and the HCPs who help them.

## Methods

### Steering group

**Figure 1:**
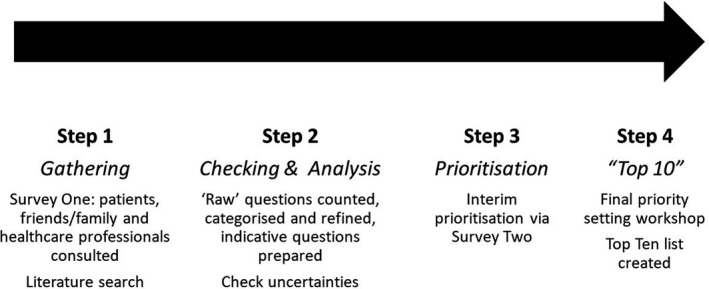
The Hyperhidrosis Priority Setting Partnership prioritization process.

A steering group was set up, comprising people with hyperhidrosis, a representative of the Hyperhidrosis UK support group, HCPs and academics, and chaired by an advisor from the James Lind Alliance. The group set the terms of reference, scope and protocol of the projected, and monitored progress throughout the various stages (Fig. 1). Established methods and best practice from the James Lind Alliance Guidebook were used.^10^

### Gathering uncertainties

An initial survey was administered online via SurveyMonkey®, which was open for 14 weeks. Participants aged ≥ 16 years, with hyperhidrosis of any severity, who were self‐diagnosed or diagnosed by an HCP, were sought via the Hyperhidrosis UK website and social media. HCPs were targeted via professional groups and societies. All contacted participants consented to take part in the PSP. The survey asked participants to suggest any questions about the treatment and management of hyperhidrosis that they would like to see answered by research, or where they felt there was uncertainty about the treatment or management of the condition. Demographic data was also collected to ensure a spread of participants from all backgrounds. This was reviewed part way through the open period, so that advertising could be targeted towards any groups that were under‐represented.

Following the initial survey responses, the steering group reviewed all submitted questions and removed any that were out of the scope of the project. Repeated or very similar questions were grouped together, and an indicative question formed to represent them (Table 1). Once the indicative questions were agreed, databases including the Cochrane Library and MEDLINE were searched to look for systematic reviews about hyperhidrosis treatments and management. A question was considered to be an ‘uncertainty’ if there was no systematic review to answer it, or if a systematic review concluded that an uncertainty existed.

**Table 1:** Indicative questions^a^ from the interim survey.

Example indicative question	Original questions
Does different clothing or footwear affect hyperhidrosis?	What to wear to help prevent sweating?
	Does wearing lighter clothing help?
	Does the type of shoe worn impact on excessive feet sweating?
	What sock and/or shoe material is best for reducing symptoms?
	Are there any good materials to wear to reduce sweatiness?
	Natural airy materials do not seem to be any better?
	Research into clothing: any info?
How safe is hyperhidrosis treatment at different stages of life, e.g. childhood, pregnancy and breastfeeding?	Are there safe treatments for children?
	What is the safest way to treat hyperhidrosis during pregnancy?
	What is the safest and most effective treatment for hyperhidrosis in children?
	Is there a treatment for hyperhidrosis, which is effective and suitable for use during pregnancy or breast feeding?

^a^ Indicative questions were formed by combining multiple similar questions together to provide one question that is representative of all of them; examples of two of the indicative questions are shown.

### Interim priority setting

A second survey listed all the indicative questions in a random order, and was open for 8 weeks. Participants were asked to select up to 10 uncertainties that they felt were the most important for research to answer. After the survey closed, the uncertainties were ranked in order of the number of times they were each selected. The uncertainties were ranked separately for HCPs and for people with hyperhidrosis and their family and friends to see whether the priorities of the two groups were different. In order to include the Top 10 priorities from each group, a total of 23 uncertainties were taken to the final workshop.

### Final workshop

Participants were invited to attend a final workshop event at De Montfort University, Leicester, UK on 30 November 2018. The participants (*n* = 18) were divided into two groups, each of which included both people with hyperhidrosis and HCPs. Each group had a trained facilitator from the James Lind Alliance who used a nominal group technique. Ground rules were set, including the need to keep discussions confidential and respect the opinions of others. The 23 questions were randomized and allocated a letter of the alphabet. Cards for each question were provided and participants worked together to rank them in order of priority. The rankings from each group were scored from 1 (the top priority) to 23 (the lowest priority), and then combined to give a total score whereby the lowest score represented the highest priority. The groups were then mixed up and given the combined rankings and the process was repeated again. Finally, a plenary session including all participants that agreed the final ranking for all 23 questions, and came up with the final Top 10 list.

## Results

### Data provision

The data that support the findings of this study are openly available in Figshare (http://doi.org/10.21253/DMU.19207539).

### Initial survey

The initial survey was completed by 268 respondents, who suggested 592 questions they would like to see answered by research. The demographic data of the respondents are shown in Table 2. Although there was a spread across all ages, 42% were in the 25–44‐year age group and 37% in the 45–64‐year age group. More women than men (79% vs. 18%) completed the survey. The majority of respondents identified as White (80%), 11% as Asian/British Asian and 4% as Black/African/Caribbean/Black British. Different groups of participants were well represented in the cohort; 58% were people with hyperhidrosis, 29% were HCPs, 11% were friends or family of people with hyperhidrosis and 2% fell into > 1 category.

**Table 2:** Participant characteristics of the participants of the first survey (*n* = 242^a^).

	*n*	%
Group		
Person with hyperhidrosis	140	58
Family or friend	27	11
Healthcare professional	70	29
In > 1 group	5	2
Sex		
Male	44	18
Female	191	79
Prefer not to say	7	3
Age		
≤ 24	36	15
25–44	102	42
45–64	90	37
≥ 65	7	3
Prefer not to say	7	3
Ethnicity		
Asian/British Asian	26	11
Black/African/Caribbean/Black British	9	4
Mixed/multiple ethnic groups	3	1
White	194	80
Other ethnic group	3	1
Prefer not to say	7	3

^a^ 26 participants did not give demographic data.

**Figure 2:**
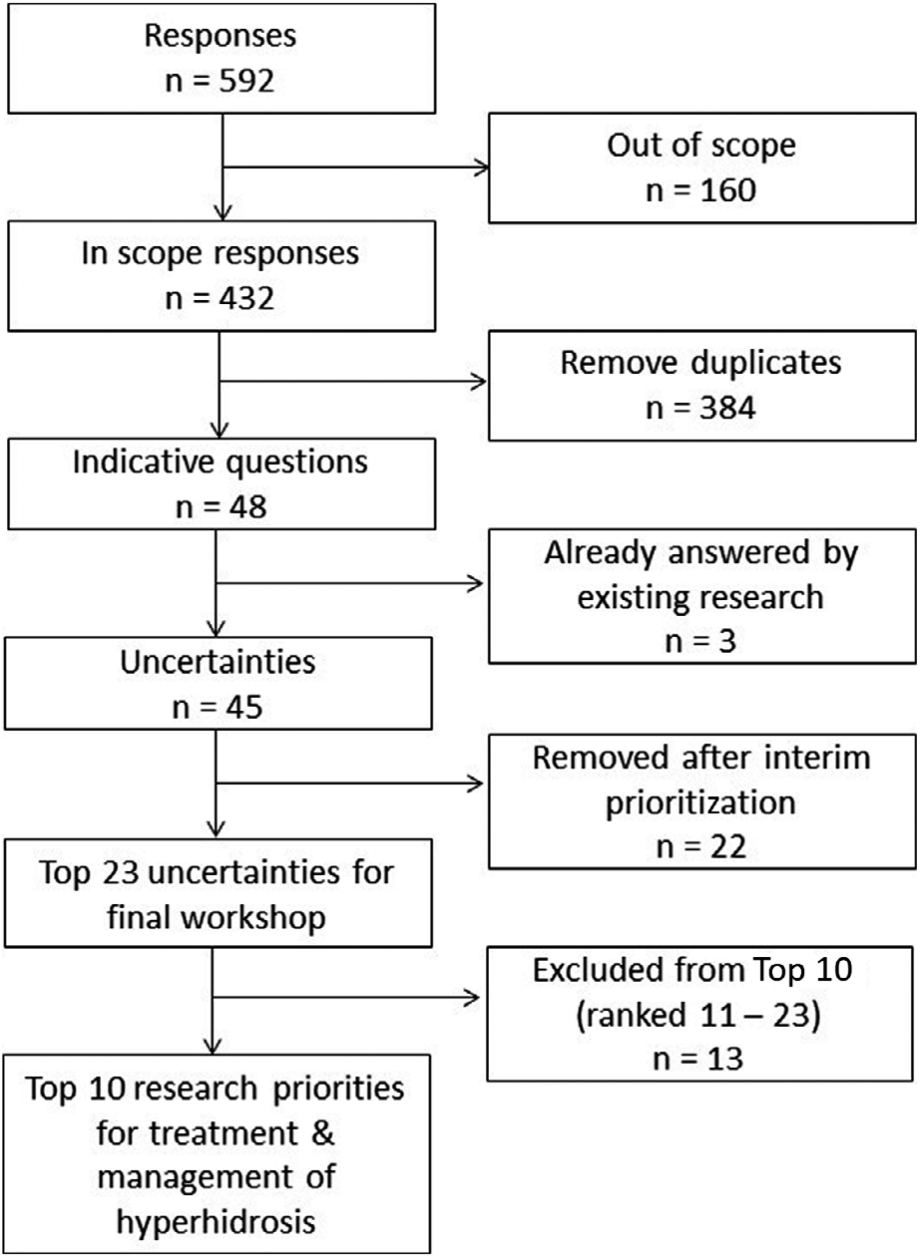
Flowchart of participant responses and prioritization.

Out of 592 questions, 160 were deemed to be out of scope (Fig. 2). The main reasons for this were individuals asking for medical advice, questions about the cause of hyperhidrosis or service provision, or questions about raising awareness of hyperhidrosis. Others were precluded because the questions were about different health conditions, or it was unclear what question was being asked. From the remaining 432 questions, 48 indicative questions were formed. Three were questions about adverse effects for which the answer is already known, leaving 45 questions that were the research uncertainties for the interim prioritization (Supplementary Table S1).

### Interim survey

The interim prioritization survey was completed by 286 participants. The demographic composition of this group was not significantly different to the participants in the first survey (data not shown). Following the interim prioritization, 23 uncertainties were selected for the final workshop. The top 23 uncertainties were ranked according to the total number of respondents who ranked the uncertainty in their Top 10. A further adjusted ranking was calculated, with the groups divided into one group comprising people with hyperhidrosis plus friends and family and a second group comprising HCPs, and given equal weightings. However, this did not change the uncertainties that were included in the top 23; instead, it made only minor changes to the ranking order.

### Final workshop

At the final workshop, the final Top 10 list was agreed (Table 3). Minor changes were agreed by consensus to the wording of questions where it was felt that the research question needed more clarity. The ranking of the remaining questions from 11–23 from the final workshop is shown in Supplementary Table S2.

**Table 3:** Top 10 research priorities for the treatment and management of hyperhidrosis.

Rank	Research priority
1	Are there any safe and effective permanent solutions for hyperhidrosis?
2	What is the most effective and safe oral treatment (drugs taken by mouth) for hyperhidrosis?
3	What are the most effective and safe ways to reduce sweating in particular areas of the body (e.g. hands, feet, underarms, face, head)?
4	How does hyperhidrosis affect quality of life?
5	Are combinations of different treatments more effective than one type of treatment for hyperhidrosis?
6	What is the most safe and effective treatment for mild to moderate hyperhidrosis?
7	Could targeted therapies or biologics (e.g. antibodies, hormones, stem cells), be effective in treating hyperhidrosis?
8	What is the most effective severity scale that can be used to determine if a person is eligible for hyperhidrosis treatment?
9	What is the safest and most effective surgery for hyperhidrosis?
10	How safe are hyperhidrosis treatments at different stages of life, e.g. childhood, pregnancy and breastfeeding?

Most of the questions in the Top 10 are related to safe and effective treatments of hyperhidrosis, while one is related to QoL and one to how hormones affect the condition. Question 8 concerned eligibility, and is most relevant in countries with government‐funded healthcare systems.

## Discussion

This PSP has highlighted the lack of evidence currently available for the treatment and management of hyperhidrosis.

The highest priority on the list was ‘Are there any safe and permanent solutions for hyperhidrosis?’. This could be answered by further work on current permanent solutions (e.g. surgery and microwave techniques); however, participants also indicated a desire for research into new treatments. The seventh priority, ‘Could targeted therapies or biologics (e.g. antibodies, hormones and stem cells), be effective in treating hyperhidrosis?’ reflects the appetite for new treatments.

Other priorities focus on whether surgery and oral treatments are effective and safe. These questions have either not been answered due to a lack of high‐quality systematic review and meta‐analysis or, more commonly, due to a lack of quality randomized clinical trials (RCTs) from which to draw data. This highlights the need for funding for more RCTs to demonstrate the safety and efficacy of treatments. A Cochrane review of hyperhidrosis was prioritized by Cochrane Skin^11^ and began in July 2021. It will attempt to answer some of the Top 10 questions, and is likely to emphasize the areas where trials are lacking.

Participants also prioritized research questions about treatments for particular areas of the body or particular life stages, and the impact of hyperhidrosis on QoL. A lack of parity over access to treatments in different areas of the UK also led to the prioritization of a question about the most effective severity scale that can be used to determine if a person is eligible for hyperhidrosis treatment; this would also have relevance in other countries with government‐funded healthcare systems.

Outside of the Top 10, there are many other important research questions to be answered, particularly in relation to the management of the condition through lifestyle changes, such as diet, exercise, clothing and footwear.

A strength of the PSP was that it had excellent support from patient groups and clinical networks, which enabled the steering group to share the surveys widely. Another strength was that all of the research questions were included in the list of indicative questions that went forward to the interim priority stage, rather than limiting the list to a selection, as has been necessary in some previous PSPs that generated very large numbers of responses.^12^, ^13^ This ensured that all voices were heard and able to be part of the priority setting.

There were also some limitations. It was not possible to confirm that all patients who took part had primary hyperhidrosis, as people were able to take part with either a self‐diagnosis or a diagnosis from a HCP. However, as the survey was promoted in support groups for people with primary hyperhidrosis and at dermatology clinics, it is probable that the majority of participants fell into this group.

Although there was good engagement from patients in the surveys, there was an issue in getting people with hyperhidrosis to engage with face‐to‐face activities, such as steering group meetings and the final workshop. This PSP was completed before the COVID‐19 pandemic, which resulted in widespread use and acceptance of online video conference meetings; such technology could have been utilized here.

Four times as many women as men completed these surveys, despite hyperhidrosis affecting women and men equally.^2^ More female participants have also been reported in other dermatology PSPs.^12^, ^14^ This is partly accounted for by 89% of registered nurses in the UK identifying as female,^15^ but also because women may be more willing to take part in health‐related surveys.^16^

An important issue raised at the final workshop was concern over lack of awareness about hyperhidrosis being a medical condition. This concern has partially been addressed since the PSP, using articles published in the media and appearances on television and radio; however, more work is needed to help people understand the impact that hyperhidrosis has on those living with this condition. Other issues raised included a lack of awareness by patients and HCPs of available treatments, and frustration over difficulties in accessing them through the National Health Service. This meant that some treatments were only available to those who could afford to be treated privately or to buy their own equipment.

## Conclusion

The Top 10 priorities from the hyperhidrosis PSP have provided a clear focus about the research areas that matter most to people with the condition and those involved in its management. They will be used to enhance research grant applications by providing evidence of patient and public involvement in partnership with HCPs in prioritizing research. However, the stories of patients that were shared during the PSP also highlighted the sense of isolation that they feel and the need for more qualitative research into hyperhidrosis involving QoL and patient‐centred outcomes as well.

## Conflict of interest

RO is an employee of Limbco Ltd, which sponsors the Hyperhidrosis UK support group website. This was declared to the steering group at the outset and he took part in the PSP purely in his capacity as a representative of the support group. The other authors declare that they have no conflict of interest.

## Supporting information


**Table S1.** Indicative questions taken forward to the interim prioritization survey. The order of questions is random.

**Table S2.** Top 11–23 research priorities for the treatment and management of hyperhidrosis from the final workshop.
