# Supplementary material for: Setting research priorities for management and treatment of hyperhidrosis: the results of the James Lind Alliance Priority Setting Partnership
Source: Clin Exp Dermatol. 2022 Mar 4;47(6):1109–14. doi: 10.1111/ced.15122 (PMC9310725; doi:10.1111/ced.15122)
Supplement: Supplementary file 1 — Table S1. Indicative questions taken forward to the interim prioritization survey. The order of questions is random. [file CED-47-1109-s002.docx]

Table S1: Indicative questions taken forward to the interim prioritisation survey. The order of questions is random.

| Q1: How does weight impact on hyperhidrosis? |
| --- |
| \| Q2: How does diet impact on hyperhidrosis? \| \| --- \| |
| Q3: Can lifestyle changes (e.g. changes to habits or routines) affect hyperhidrosis? |
| Q4: How does hyperhidrosis affect quality of life? |
| Q5: Does regular exercise affect hyperhidrosis? |
| Q6: Do different clothing or footwear materials affect hyperhidrosis? |
| Q7: How do products such as soaps, moisturisers or alcohol-based wipes affect hyperhidrosis? |
| Q8: Does laser hair removal affect the amount of sweating? |
| Q9: How does hyperhidrosis vary with age? |
| Q10: Is hyperhidrosis more common in specific ethnic groups? |
| Q11: How should management and/or treatment of hyperhidrosis be different for children and adults? |
| Q12: How does gender affect hyperhidrosis? |
| Q13: Are there any other health conditions (e.g. high blood pressure, fibromyalgia, eczema etc.) that are linked to primary hyperhidrosis (hyperhidrosis with no currently known underlying cause)? |
| Q14: How do hormones (e.g. puberty, menstrual cycle or hormonal imbalances) affect people with hyperhidrosis? |
| Q15: How safe are hyperhidrosis treatments at different stages of life, e.g. childhood, pregnancy and breastfeeding? |
| Q16: What is the most effective and safe way to reduce sweating on the face and head? |
| Q17: What are the most effective and safe ways to reduce sweating in particular areas of the body (e.g. hands, feet or underarms)? |
| Q18: How safe and effective is Miradry (electromagnetic treatment) compared to other hyperhidrosis treatments? |
| Q19: Are there long term side effects of Miradry (electromagnetic treatment)? |
| Q20: How safe and effective is iontophoresis compared to other treatments? |
| Q21: What is the best liquid solution to use (e.g. water or water plus additives) for iontophoresis treatment? |
| Q22: What is the best way to administer iontophoresis? E.g. electric current, pattern of treatment, and use at home or in clinic. |
| Q23: How does iontophoresis work? |
| Q24: How effective is iontophoresis for parts of the body other than hands and feet? |
| Q25: How safe and effective are antiperspirants for hyperhidrosis? |
| Q26: What is the best way to reduce the side effects of antiperspirants? |
| Q27: What are the best ways to control the odour from hyperhidrosis? |
| Q28: Are combinations of different treatments more effective than one type of treatment for hyperhidrosis? |
| Q29: How safe and effective is Botox compared to other hyperhidrosis treatments? |
| Q30: What is the best way to administer Botox to people with hyperhidrosis? E.g. pain relief, depth of injection, frequency of treatment etc.) |
| Q31: Do different groups of people with hyperhidrosis respond differently to Botox? |
| Q32: What is the most effective way to determine whether a person is eligible for hyperhidrosis treatments? |
| Q33: What are the long-term effects of sweat gland removal for hyperhidrosis? |
| Q34: What are the long-term effects of endoscopic thoracic sympathectomy for hyperhidrosis? |
| Q35: What is the most effective surgery for hyperhidrosis? |
| Q36: What is the most effective treatment for mild to moderate hyperhidrosis? |
| Q37: Are there any safe and effective permanent solutions for hyperhidrosis? |
| Q38: What is the most effective and safe oral treatment (drugs taken by mouth) for hyperhidrosis? |
| Q39: Could gene therapy be used to help treat hyperhidrosis? |
| Q40: Could targeted therapies or biologics (e.g. antibodies, hormones, stem cells), be effective in treating hyperhidrosis? |
| Q41: Could a vaccine be created to prevent hyperhidrosis? |
| Q42: Is physiotherapy an effective treatment for hyperhidrosis? |
| Q43: Are there any links between hyperhidrosis and mental health (e.g. anxiety, depression)? |
| Q44: Can psychological, talking or educational interventions help treat people with hyperhidrosis? |
| Q45: How do complementary therapies (e.g. meditation, hypnosis, acupuncture etc.) affect hyperhidrosis? |
