# Supplementary material for: Setting research priorities for management and treatment of hyperhidrosis: the results of the James Lind Alliance Priority Setting Partnership
Source: Clin Exp Dermatol. 2022 Mar 4;47(6):1109–14. doi: 10.1111/ced.15122 (PMC9310725; doi:10.1111/ced.15122)
Supplement: Supplementary file 2 — Table S2. Top 11–23 research priorities for the treatment and management of hyperhidrosis from the final workshop. [file CED-47-1109-s001.docx]

Table S2: Top 11-23 research priorities for the treatment and management of hyperhidrosis from the final workshop

| **Rank** | **Research priority** |
| --- | --- |
| 11 | How do hormones (e.g. puberty, menstrual cycle or hormonal imbalances) affect people with hyperhidrosis? |
| 12 | Are there links between hyperhidrosis and mental health (e.g. anxiety, depression)? |
| 13 | Are there any other health conditions (e.g. high blood pressure, fibromyalgia, eczema etc.) that are linked to primary hyperhidrosis (hyperhidrosis with no currently known underlying cause)? |
| 14 | What is the most effective and safe way to reduce sweating on the face and head? |
| 15 | How safe and effective are antiperspirants for hyperhidrosis? |
| 16 | Can lifestyle changes (e.g. changes to habits or routines) affect hyperhidrosis? |
| 17 | What are the long-term effects of sweat gland removal for hyperhidrosis? |
| 18 | How does diet impact on hyperhidrosis? |
| 19 | What are the best ways to control the odour from hyperhidrosis? |
| 20 | How do complementary therapies (e.g. meditation, hypnosis, acupuncture etc.) affect hyperhidrosis? |
| 21 | How do products such as soaps, moisturisers or alcohol-based wipes affect hyperhidrosis? |
| 22 | How does weight impact on hyperhidrosis? |
| 23 | Could a vaccine be created to prevent hyperhidrosis? |
